# The polysaccharide from *Camellia oleifera* fruit shell enhances immune responses via activating MAPKs and NF-κB signaling pathways in RAW264.7 macrophages

**DOI:** 10.29219/fnr.v66.8963

**Published:** 2022-12-05

**Authors:** Chuanqi Xie, Xinying Lin, Juwu Hu, Shufen Wang, Jing Wu, Wei Xiong, Lei Wu

**Affiliations:** 1 Institute of Applied Chemistry, Jiangxi Academy of Sciences, Nanchang, P.R. China; 2 School of Medicine, Xiamen University, Xiamen, China

**Keywords:** C. oleifera *fruit shell polysaccharide*, *RAW264. 7 macrophage*, *immunoregulatory activities*, *MAPKs*, *NF-*κ*B*

## Abstract

**Background:**

Macrophage plays an important role in innate immune responses by secreting immune molecules and phagocytosis. *Camellia oleifera* fruit shell, accounting for approximately 60% weight of the single *C. oleifera* fruit, is rich in polysaccharides and has several biological activities such as anti-oxidation, lipid regulation and anticancer. However, the immunomodulatory activity of the polysaccharide from *C. oleifera* fruit shells (CPS) has not been reported.

**Objective:**

This study aimed to investigate the immunomodulatory activities and mechanisms of CPS in RAW264.7 macrophages.

**Methods:**

The Methyl Thiazolyl Tetrazolium (MTT) method was used to evaluate the effect of CPS on the cell viability of RAW264.7 macrophages, and cell morphology was pictured using microscope. The production of immune-related molecules, including nitric oxide (NO), prostaglandin E2 (PGE2), tumor necrosis factor α (TNFα), interleukin (IL)-1β and IL-6, was detected by Griess assay and enzyme-linked immunosorbent assay (ELISA). The protein expression of inducible nitric oxide synthase (iNOS) and cyclooxygenase 2 (COX2) and the phosphorylation level of mitogen-activated protein kinases (MAPKs) were analyzed through western blotting. The mRNA levels of related genes were tested using reverse transcription-polymerase chain reaction (RT-PCR). The nuclear translocation of nuclear factor-kappa B (NF-κB) was detected using immunofluorescence technology.

**Results:**

The results indicated that CPS treatment stimulated the production of NO and PGE2 and significantly enhanced the protein expression of iNOS and COX2 with little effect on the cell morphology and viability. Also, the secretion and mRNA levels of TNFα were increased by the treatment of CPS. In addition, CPS treatment markedly upregulated the phosphorylation level of MAPKs including Extracellular Signal Regulated Kinase (ERK), P38, and c-Jun N-terminal Kinase (JNK) at different time points and caused the activation and nuclear translocation of NF-κB.

**Conclusion:**

Our data implied that CPS exerts immunomodulatory activities by activating MAPKs and NF-κB signaling pathways in RAW264.7 macrophages.

## Popular scientific summary

- The polysaccharide from *Camellia oleifera* fruit shell (CPS) promoted the production of nitric oxide (NO) and prostaglandin E2 (PGE2) in RAW264.7 macrophages.
- CPS significantly enhanced the protein expression of inducible nitric oxide synthase (iNOS) and cyclooxygenase 2 (COX2) in RAW264.7 macrophages.
- CPS increased the secretion and mRNA levels of tumor necrosis factor α (TNFα) in RAW264.7 macrophages.
- CPS markedly upregulated the phosphorylation level of mitogen-activated protein kinases (MAPKs) and promoted the activation and nuclear translocation of nuclear factor-kappa B (NF-κB) in RAW264.7 macrophages.

Macrophages exist in all tissues are a kind of immune cells, which play an important role in the host immune response to the invasion of pathogenic microorganisms and other foreign substances by secreting immune molecules and related cytokines such as nitric oxide (NO), prostaglandin E2 (PGE2), tumor necrosis factor α (TNFα), interleukin (IL)-1β, and IL-6 (1–3). NO and PGE2 are two significant immune molecules involved in a variety of physiological and pathological reactions in the host (4, 5). TNFα, IL-1β, and IL-6 are three typical cytokines regulating immune responses in macrophages, which are considered the marked mediators in several cell processes, including immune reaction, inflammatory response, cell proliferation, and cell apoptosis (6–9).

Mitogen-activated protein kinases (MAPKs) and nuclear factor-kappa B (NF-κB) are two major signaling pathways related to the activation of macrophages, which regulate the secretion and maturation of immune molecules and related cytokines in macrophages. MAPKs are serine/threonine protein kinases presented in most eukaryotes, containing three family members including P38, ERK, and JNK, which can be activated by the transforming growth factor-β (TGF-β)-activated kinase 1 (TAK1) (3, 10). The activation of MAPKs then promotes the nuclear translocation of activation protein 1 (AP1) from cytoplasm, which regulates mRNA transcription via binding to the promoter region of related genes and ultimately causes the secretion and maturation of related cytokines and immune molecules such as inducible nitric oxide synthase (iNOS), cyclooxygenase 2 (COX2), TNFα, IL-1β, and IL-6 (11–14). NF-κB is an important transcription factor that regulates a series of immune responses by promoting the release of related molecules and enhancing the phagocytosis ability of macrophages (15). NF-κB in the rest state remains in the cytoplasm by binding to the IκB inhibitory protein. Once the cells are stimulated by a foreign substance or pathogen, the IκB inhibitory protein will be phosphorylated by the IκB kinase (IKK) and dissociated from the NF-κB complex and then degraded through ubiquitination. The dissociated NF-κB will be activated and rapidly translocated into nuclear to regulate the transcription of related genes by binding to the mRNA promoter region, ultimately leading to the release of related molecules and the activation of macrophages (16, 17).

*C. oleifera* fruit shell, accounting for approximately 60% weight of the single *C. oleifera* fruit, is rich in polysaccharides and has several biological activities such as anti-oxidation, lipid regulation, and anticancer. Xie and colleagues have pointed out that the extracts of *C. oleifera* fruit shell could scavenge free radicals, inhibit lipid peroxidation, or reduce the body weight of mice induced by high fat diet (18). Jin et al. and Zhang et al. (19, 20) have shown that the polysaccharide from *C. oleifera* fruit shell (CPS) had promising antitumor activity as well as strong antioxidant activity in vitro and in vivo. However, the immune activity of the polysaccharides extracted from *C. oleifera* fruit shells (CPS) has not been reported.

In this study, the effects of CPS on immune responses of RAW264.7 macrophages and the underlying mechanisms were evaluated. The results indicated that CPS treatment significantly promoted the production of NO and PGE2 and enhanced the protein expression of iNOS and COX2 with little effect on the cell morphology and viability. Moreover, the secretion and mRNA level of TNF-α were increased by the treatment of CPS. Mechanically, CPS treatment markedly upregulated the phosphorylation level of MAPKs including ERK, P38, and JNK at different time points and promoted the activation and nuclear translocation of NF-κB. In conclusion, our data implied that CPS exerted immunomodulatory activities in RAW264.7 macrophages by activating MAPKs and NF-κB signaling pathways.

## Materials and methods

### The source of polysaccharides extracted from C. oleifera fruit shell

In this study, *C. oleifera* fruit shell was provided by Jiangxi enquan grease Co., Ltd (Shangrao, China). The polysaccharide was extracted from *C. oleifera* fruit shell by water extraction at 100°C for 74.5 min with solid-water ratio of 1:21 g/mL. It was then precipitated with 80% ethanol, followed by decolorization with 25% activated carbon. Finally, the content of polysaccharide was determined using the phenol-sulfuric acid method, and the content of CPS used in this study increased to 34.93%.

### Cell culture

RAW264.7 macrophage was a gift from Key Laboratory of Pu-er Tea Science, Ministry of Education, which was cultured in high glucose DMEM media (Biological Industries, Israel) supplemented with 10% fetal bovine serum (Natocor, Argentina) and 1% penicillin and streptomycin (Biological Industries, Israel) at 37°C with 5% CO_2_. The cells were observed every day, and the culture medium was changed every 2~3 days.

### MTT assay

RAW264.7 cells in logarithmic growth phase were seeded in a 96-well plate (5 × 10^4^ cells/well) and incubated overnight. CPS at a concentration of 25, 50, 100, or 200 µg/mL or Lipopolysaccharide (LPS) (Sigma-Aldrich, USA) at 1 µg/mL was added to the plant and incubated for another 24 h. Then, MTT (Solarbio, Beijing) reagent was supplemented to each well for 4 h at 37°C under the dark condition. Supernatants were entirely aspirated, and Dimethylsulfoxide (DMSO) were added to dissolve the formazan crystals. After shaking for 10 min, the absorbance was measured at 492 nm using a microplate reader (Tecan Infinite 200 Pro, Switzerland).

### Griess assay

RAW264.7 cells (5 × 10^4^ cells/well) were inoculated in a 96-well plate overnight and then treated with CPS or LPS for 24 h. The production of NO was measured by the accumulation of nitrites in the culture medium, using the colorimetric Griess reaction with slight modifications. 100 µL of each supernatant medium was mixed with an equal volume of Griess A (0.1% [w/v] N-(1-naphthyl)-ethylenediamine dihydrochloride) and B (1% [w/v] sulphanilamide containing 5% [w/v] H_3_PO_4_) (1:1) at room temperature for 10 min in the dark. The absorbance was immediately measured at 540 nm, and the concentration of NO was calculated by the standard curve of nitrite.

### Enzyme-linked immunosorbent assay

RAW264.7 cells were seeded in a 6-well plate at a density of 1 × 10^6^ cells/well and cultured overnight. Drugs were added in the plate and incubated for 24 h. The supernatants were centrifuged at 15,000 g, 4°C for 10 min and collected for the detection of related immunoregulatory molecules using the enzyme-linked immunosorbent assay (ELISA) method. The contents of TNFα, IL-1β, and IL-6 were determined according to the instructions from the manufacturer of ELISA kit (BIOSTER, Wuhan, China).

### Western blotting

RAW264.7 cells (1 × 10^6^ cells/well) were treated with the same way of ELISA or inoculated in dishes with a diameter of 6 cm overnight and stimulated with CPS or LPS for the indicated times, respectively. Then, cells were lysed in radioimmunoprecipitation assay (RIPA) lysis buffer (Solarbio, Beijing, China) on ice for 30 min, and proteins were extracted and quantified by BCA Kit (Beyotime, Jiangsu, China). 40 µg of protein was separated via 8 or 10% sodium dodecyl sulfate (SDS)-polyacrylamide gel and then transferred onto Polyvinylidene Fluoride (PVDF) membranes (Millipore, CA, USA). The membranes were blocked with 5% Bovine Serum Albumin (BSA) or skim milk at room temperature for 1 h and then incubated with primary antibodies specific for iNOS, ERK1/2, pERK1/2, P38, and JNK1/2/3 (ABclonal, Wuhan, China), and COX2, Glyceraldehyde-3-Phosphate Dehydrogenase (GAPDH), p-P38, and pJNK (Bioworld Technology, Inc, MN, USA) at 4°C overnight. The membranes were then incubated for an additional 1 h with goat antirabbit lgG/ Horseradish Peroxidase (HRP) (Bioss, Beijing, China). Then, the membrane was developed using HyperSignal ECL Plus kit (4A Biotech, Beijing, China) for imaging with ChemiScope 3000 mini (Clinx, Shanghai, China).

### RNA extraction and quantitative real-time PCR (qRT-PCR) experiment

RAW264.7 cells (1 × 10^6^ cells/well) were treated with the same way of ELISA and lysed in TransZol Up reagent (TransGen Biotech, Beijing, China). RNA was extracted according to the instructions from the manufacturer and quantified with a microplate reader (Tecan Infinite 200 Pro, Switzerland). cDNA was synthesized with Primescript™ First-Strand cDNA Synthesis kit (Takara, Beijing, China). The real-time polymerase chain reaction (PCR) was performed using qTOWER 3G (Analytic Jena, Jena, Germany) with TB Green® Premix Ex Taq™ II kit (Takara, Beijing, China). The obtained amplification data were calculated using GAPDH as internal reference. The specific primers are shown in Table 1.

**Table 1:** qRT-PCR primer sequences

Primer name	Primer sequences
iNOS-F	CCCTTCCGAAGTTTCTGGCAGCAGC
iNOS-R	GGCTGTCAGAGCCTCGTGGCTTTG
COX2-F	AGAAGGAAATGGCTGCAGAA
COX2-R	GCTCGGCTTCCAGTATTGAG
TNFα-F	TGTCCCTTTCACTCACTGGC
TNFα-R	CATCTTTTGGGGGAGTGCCT
IL-1β-F	AGCTTCAGGCAGGCAGTATC
IL-1β-R	AAGGTCCACGGGAAAGACAC
IL-6-F	GAGTGGCTAAGGACCAAGACC
IL-6-R	AACGCACTAGGTTTGCCGA
GAPDH-F	CACTCACGGCAAATTCAACGGCA
GAPDH-R	GACTCCACGACATACTCAGCAC

### NF-κB activation and nuclear translocation assay

RAW264.7 cells (1 × 10^5^ cells/well) seeded in a 6-well plate were treated with CPS or LPS for 6 h. Then, the supernatants were discarded, and the immunofluorescence test was performed according to the instructions from the manufacturer of NF-κB activation nuclear translocation assay Kit (Beyotime, Jiangsu, China). Cells were fixed with the stationary liquid for 15 min and blocked with blocking solution for 1 h at room temperature. The cells were then incubated overnight at 4°C with the primary antibody NF-κB. Following washing, the NF-κB antibody was recycled and further incubated with Cy3-conjugated secondary antibody for 1 h and stained with 4′, 6-diamidino-2-phenylindole (DAPI) for 5 min. Images were captured using a TS2-FL microscope (Nikon, Tokyo, Japan).

### Statistical analysis

Three replicates were performed for all experiments, and the results were expressed as mean ± standard deviation. The statistical test was performed using GraphPad Prism, version 8.0.1 (GraphPad Software, San Diego, CA, USA), and the significance analysis was performed by t-test.

## Results

### Effects of CPS on the cell morphology and viability of RAW264.7 macrophages

**Fig. 1:**
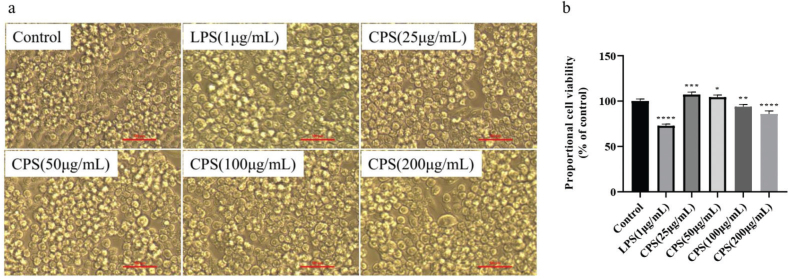
Effects of CPS on the cell morphology and viability of RAW264.7 macrophages. Cells were treated with indicated concentrations of CPS (0, 25, 50, 100, and 200 µg/mL) and LPS (1 μg/mL) for 24 h. The cell morphology was pictured by microscope (a). The cell viability was evaluated by MTT reagent (b). The data were presented as means ± SD of three separate experiments. *P < 0.05, **P < 0.01, ***P < 0.001, and ****P < 0.0001 compared with control group.

In this study, CPS was obtained from *C. oleifera* fruit shells by hot water extraction, 80% ethanol precipitation, and 25% activated carbon adsorption. The effects of CPS on the cell morphology and viability of RAW264.7 macrophages were evaluated through microscope and MTT assay. The results showed that CPS treatment had little effect on the cell morphology, although LPS treatment resulted in morphological changes (Fig. 1a). And the cell viability of RAW264.7 macrophages in CPS treated group was higher than LPS treated group, which was increased first and then decreased with the increase of CPS concentration (Fig. 1b). These results suggested that CPS has less cytotoxicity.

### Effects of CPS on the production of NO and PGE2 in RAW264.7 macrophages

**Fig. 2:**
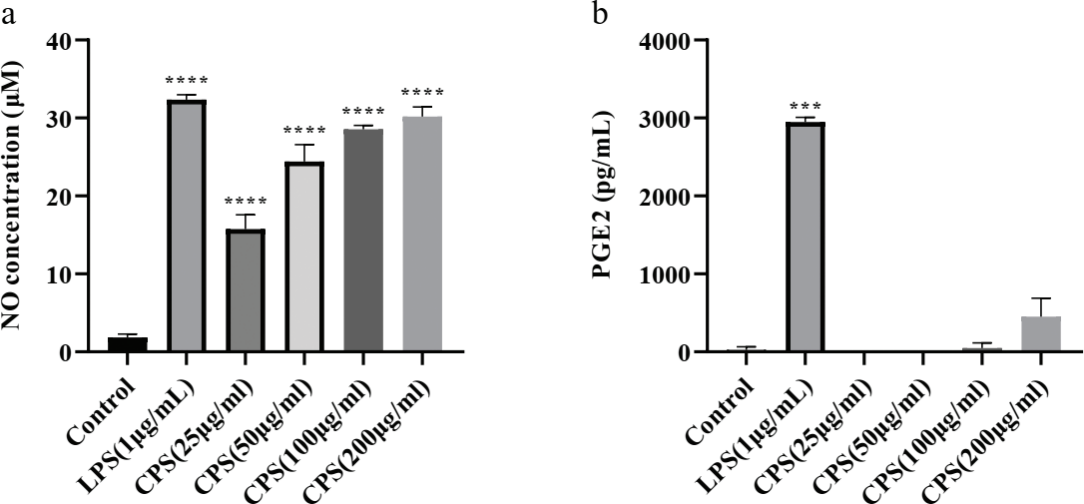
Effects of CPS on the production of NO and PGE2 in RAW264.7 macrophages. Cells were treated with indicated concentrations of CPS (0, 25, 50, 100, and 200 µg/mL) and LPS (1 μg/mL) for 24 h. The cell supernatants were collected, and the production of NO was detected by Griess assay (a). The PGE2 production was determined by ELISA (b). The data were presented as means ± SD of three separate experiments. ***P < 0.001, ****P < 0.0001 compared with control group.

NO and PGE2 are two signal molecules produced by macrophages in the activation process. And the release amount of NO and PGE2 indicates the activation degree of macrophages in immune stimulation. The effects of CPS on the production of NO and PGE2 were detected by Griess method and ELISA. The results demonstrated that CPS significantly promoted the production of NO in a dose-dependent manner in RAW264.7 macrophages, and LPS as a positive control induced the production of NO (Fig. 2a). Moreover, the release of PGE2 was gradually increased with the increase of CPS concentration, and at the same time, LPS treatment stimulated the release of PGE2 (Fig. 2b). These results indicated that CPS played a regulatory role in the production of immune molecules in RAW264.7 macrophages.

### Effects of CPS on the protein expression of iNOS and COX2 in RAW264.7 macrophages

**Fig. 3:**
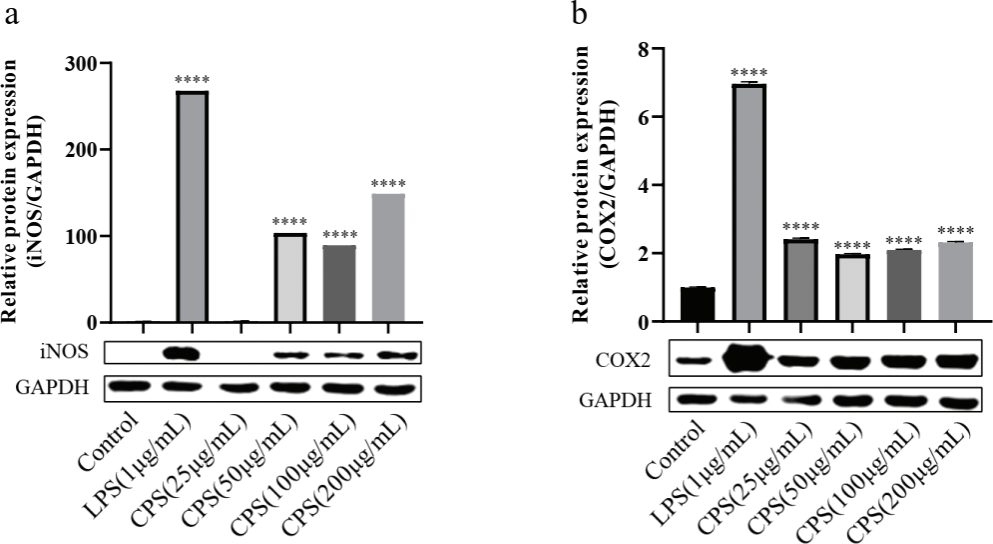
Effects of CPS on the protein expression of iNOS and COX2 in RAW264.7 macrophages. Cells were treated with indicated concentrations of CPS (0, 25, 50, 100, and 200 µg/mL) and LPS (1 μg/mL) for 24 h. The cell lysates were obtained, and the protein expression of iNOS (a) and COX2 (b) was detected by western blotting. The data were presented as means ± SD of three separate experiments. ****P < 0.0001 compared with control group.

iNOS and COX2 are two key enzymes responsible for the synthesis of NO and PGE2 in RAW264.7 macrophages with immune stimulation. In this study, the effects of CPS on the protein expression of iNOS and COX2 were analyzed by western blotting. As shown in Fig. 3, CPS treatment significantly upregulated the protein expression of iNOS and COX2, even though LPS induced the protein expression of iNOS and COX2 in a higher level.

### Effects of CPS on the secretion of immunomodulatory factors in RAW264.7 macrophages

**Fig. 4:**
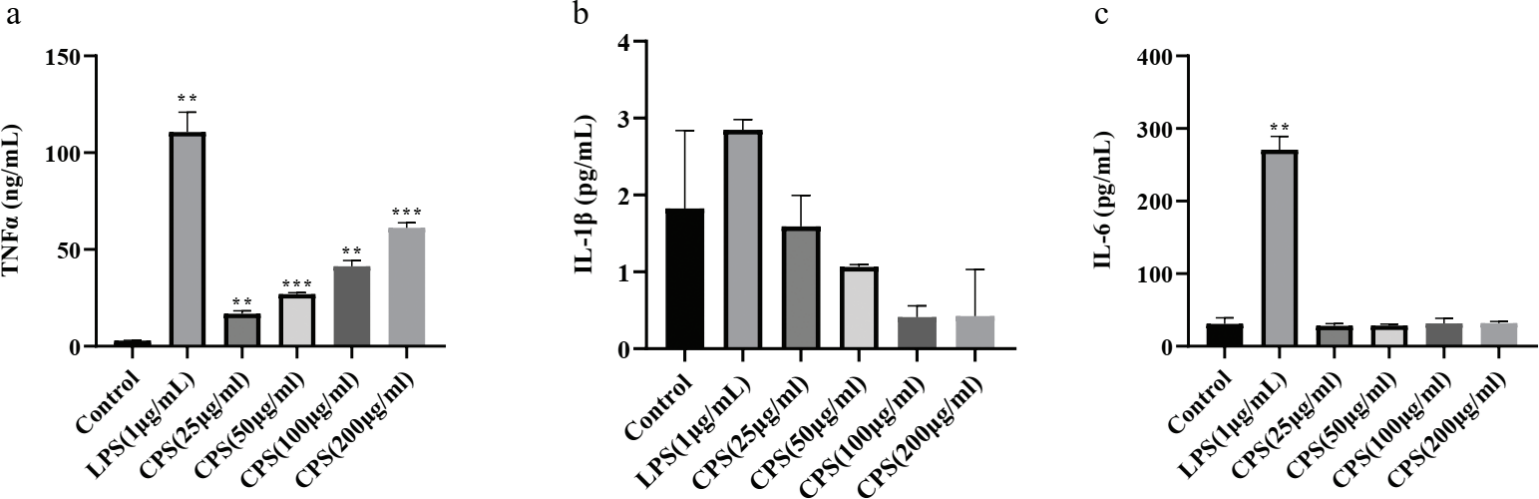
Effects of CPS on the secretion of TNFα, IL-1β, and IL-6 in RAW264.7 macrophages. Cells were treated with indicated concentrations of CPS (0, 25, 50, 100, and 200 µg/mL) and LPS (1 μg/mL) for 24 h. The cell supernatants were collected, and the secretion of TNFα (a), IL-1β (b), and IL-6 (c) was detected by ELISA. The data were presented as means ± SD of three separate experiments. **P < 0.01, ***P < 0.001 compared with control group.

The secretion of immunomodulatory factors is closely related to the degree of immunological response in macrophages. TNFα, IL-1β, and IL-6 are three major immunomodulatory factors in macrophages stimulated by immunogens. Here, the effects of CPS on the secretion of TNFα, IL-1β, and IL-6 in the cell supernatant of RAW264.7 macrophages were detected using the ELISA method. The results displayed that CPS treatment dramatically increased the secretion of TNFα in a dose-dependent manner, although LPS stimulated the secretion of TNFα in a higher level (Fig. 4a). In addition, the secretion of IL-1β and IL-6 was not promoted by the treatment of CPS (Fig. 4b, c). These data suggested that the immunomodulatory activity of CPS may be related to the increase of TNFα secretion in RAW264.7 macrophages.

### Effects of CPS on the mRNA level of immune-related molecules in RAW264.7 macrophages

**Fig. 5:**
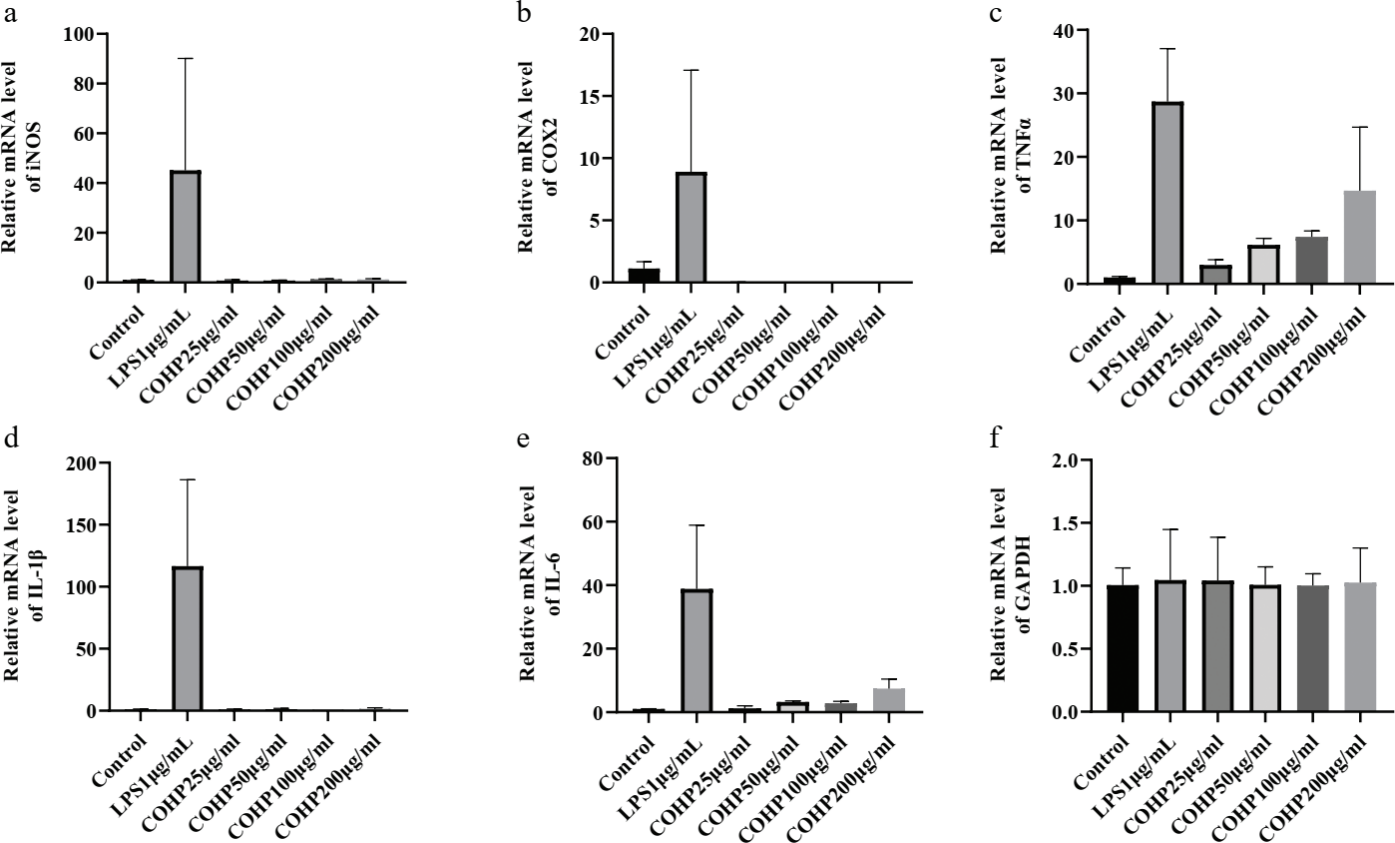
Effects of CPS on the mRNA level of iNOS, COX2, TNFα, IL-1β, and IL-6 in RAW264.7 macrophages. Cells were treated with indicated concentrations of CPS (0, 25, 50, 100, and 200 µg/mL) and LPS (1 μg/mL) for 24 h. The cell lysates were collected for RNA extraction, and the mRNA levels of iNOS (a), COX2 (b), TNFα (c), IL-1β (d), IL-6 (e), and GAPDH (f) were analyzed using RT-PCR. The data were presented as means ± SD of three separate experiments.

To further evaluate the immunomodulatory activity of CPS, the mRNA levels of immune-related molecules in RAW264.7 macrophages treated with CPS were detected using the RT-PCR method. As presented in Fig. 5, CPS treatment upregulated the mRNA level of TNFα in a dose-dependent manner but had little effect on the mRNA levels of iNOS, COX2, IL-1β, and IL-6 in RAW264.7 macrophages. LPS treatment induced the upregulation of the mRNA levels of all immune-related molecules. And GAPDH was as the internal reference. These results indicated that CPS might play the immunomodulatory activity via upregulating the mRNA level of TNFα.

### CPS regulated the phosphorylation of MAPK signaling pathway in RAW264.7 macrophages

**Fig. 6:**
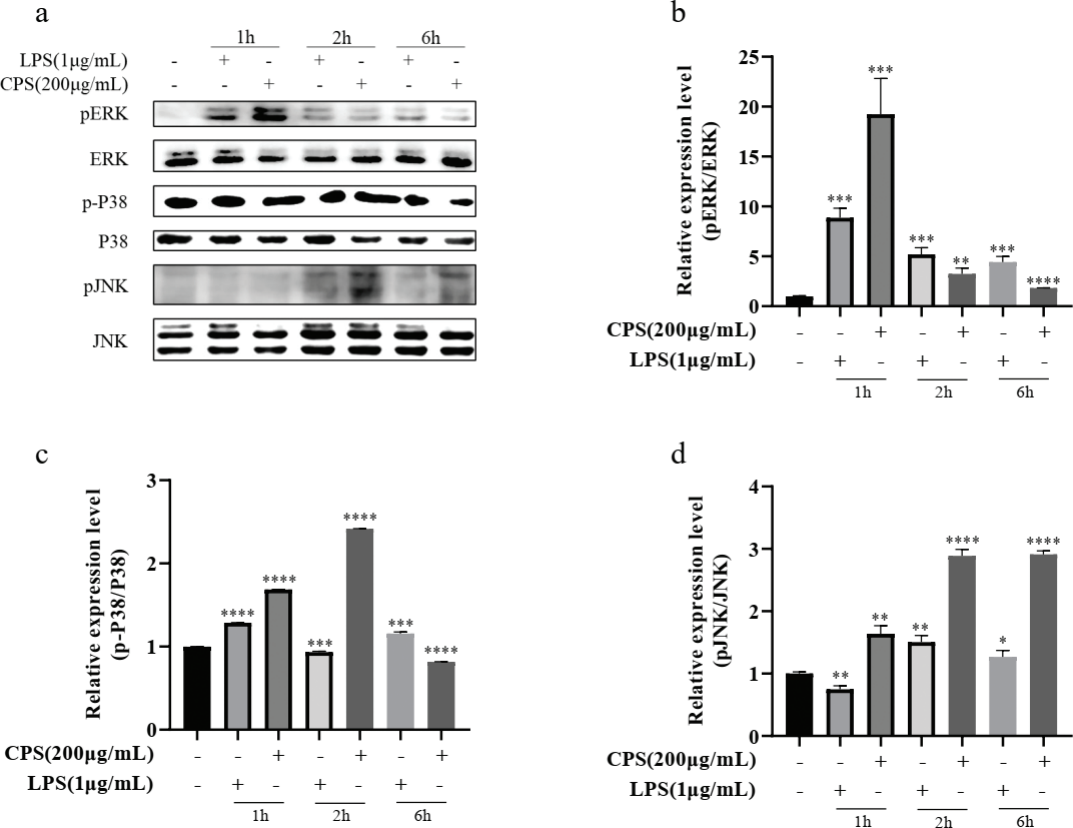
Effects of CPS on the phosphorylation of MAPKs in RAW264.7 macrophages. Cells were treated with CPS (200 µg/mL) and LPS (1 μg/mL) for the indicated times. The cell lysates were obtained, and the protein expression and phosphorylation of ERK, P38, and JNK were detected by Western blotting. (a) Representative results of western blotting bands. (b-d) The relative phosphorylation levels of ERK (b), P38 (c), and JNK (d) were quantified using image J and normalized to GAPDH. The data are presented as means ± SD of three independent experiments. **P < 0.01, ***P < 0.001, ****P < 0.0001 compared with control group.

MAPK signaling pathway plays a significant regulatory role in the immune responses of macrophages. In this study, to evaluate the immune regulatory mechanism of CPS, the activation of MAPKs, including the phosphorylation of ERK, P38, and JNK in RAW264.7 macrophages, was analyzed using western blotting. The results demonstrated that the phosphorylation of ERK was activated by the treatment of CPS, which increased to the highest level at 1 h and then decreased gradually at 2 and 6 h (Fig. 6a, b). Moreover, CPS treatment upregulated the phosphorylation level of p38 and JNK at different time points (Fig. 6a, c and d). These data suggested that CPS activated the phosphorylation of MAPK signaling pathway in a time-dependent manner in RAW264.7 macrophages.

### CPS caused the nuclear translocation of NF-κB in RAW264.7 macrophages

**Fig. 7:**
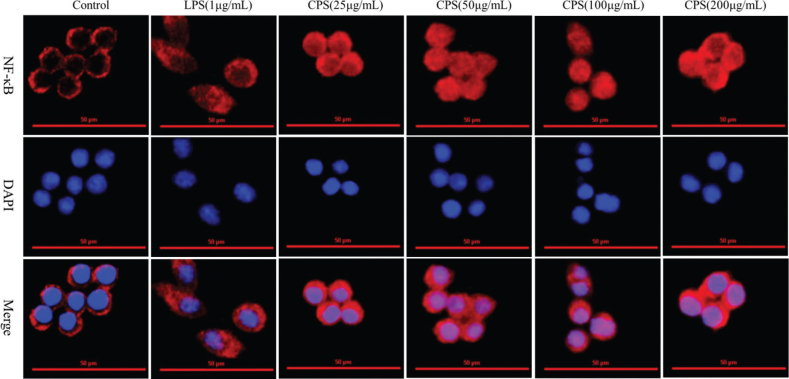
Effects of CPS on the nuclear translocation of NF-κB in RAW264.7 macrophages. Cells were treated with indicated concentrations of CPS (0, 25, 50, 100, and 200 µg/mL) and LPS (1 μg/mL) for 6 h. The cell supernatants were discarded, and the cells were used for the detection of NF-κB nuclear translocation via immunofluorescence. The representative graphs of immunofluorescence were presented from three separate experiments.

NF-κB is a classical signaling pathway involved in the regulation of immune and inflammatory responses in macrophages. Here, the nuclear translocation and activation of NF-κB in RAW264.7 macrophages was examined using immunofluorescence technique to explore the mechanism that CPS played an immunomodulatory activity. As the result shown in Fig. 7, the red fluorescence demonstrated that NF-κB remained in the cytoplasm in RAW264.7 macrophages in the resting state and then transferred into the nuclear after treated by CPS at different concentrations for 6 h. LPS was as the positive control, which also promoted the nuclear translocation of NF-κB. This result suggested that CPS treatment caused the activation and nuclear translocation of NF-κB in RAW264.7 macrophages.

## Discussion

Several researches have reported that polysaccharides existing widely in living organisms have the ability to regulate immunity by activating macrophages, which play a significant role in host defense against invasion of foreign microorganisms and other pathogens by phagocytosis and secreting several cytokines, including iNOS, PGE2, TNFα, IL-1β, and IL-6 (21–24). Jing and colleagues showed that the polysaccharide derived from *Ligustrum lucidum* can improve the immune function of immunosuppressed animals, increase the ratio of macrophages, and significantly improve the phagocytic percentage and phagocytic index of peritoneal macrophages in immunosuppressed mice to chicken red blood cells (25). Ji et al. (26) reported that the polysaccharide from *Atractylodes macrocephala Koidz* can stimulate macrophages and promote the production of NO and TNFα. Lee et al. (27) pointed out that the polysaccharide extracted from the *Korean mulberry* fruit oddi can activate RAW264.7 cells and promote the release of TNFα and IL-6, which can be used as an effective immune modulator. However, the immunomodulatory activity of the CPS has not been elucidated. In this study, the effects of CPS on the activation of RAW264.7 macrophages were investigated via Griess, ELISA, western blotting, RT-PCR, immunofluorescence technology, etc.

NO and PGE2, as two important immune molecules synthesized and secreted by macrophages, were mainly derived from L-arginine and arachidonic acid by the action of iNOS and COX2, respectively (28). When the macrophages were stimulated by a foreign substance, the production of NO and PGE2 will be significantly improved. In our results, the production of NO and PGE2 and the protein expression of iNOS and COX2 were increased by the treatment of CPS (Figs. 1 and 2). However, the mRNA levels of iNOS and COX2 were not improved by the treatment of CPS (Fig. 4a, b). These results suggested that the effect of CPS on the expression of iNOS and COX2 may be at the translation level but not the transcription level.

Moreover, macrophages in the activated state can also produce a variety of immune-related cytokines such as TNF-α, IL-1β, and IL-6, which are considered the major mediators in several cell processes, including immune reaction, inflammatory response, cell proliferation, and cell apoptosis (6–9, 29). In this study, our data indicated that CPS treatment induced the secretion of TNFα and improved its mRNA level in RAW264.7 macrophages, but the release of IL-1β and IL-6 and their mRNA levels were not increased by the treatment of CPS. These results indicated that the immune activity of CPS on RAW264.7 macrophages may be closely related to the release of TNFα, but not IL-1β and IL-6.

In addition, MAPKs and NF-κB signaling pathways are closely related to the activation of macrophages stimulated by polysaccharides, which can regulate the secretion and maturation of immune molecules and related cytokines in macrophages (30). Xu and colleagues pointed out that *Pleurotus eryngii* polysaccharide can induce the secretion of immune-related cytokines and promote the phosphorylation of JNK, ERK, and P38 in RAW264.7 cells (31). Long et al. (32) reported that *Polygonatum sibiricum* polysaccharide can trigger MAPK and NF-κB pathways, produce cytokines, and activate JNK, ERK, and P38 proteins. Our results demonstrated that CPS treatment significantly promoted the phosphorylation levels of ERK, JNK, and P38 in RAW264.7 macrophages at different time points. The activation and nuclear translocation of NF-κB were regulated by the treatment of CPS. These data implied that the effects of CPS on immune responses of RAW264.7 macrophages were achieved through activating MAPK and NF-κB signaling pathways.

## Conclusion

In this study, the immunoregulatory activities and underlying mechanisms of CPS in RAW264.7 macrophages were investigated. The data indicated that CPS increased the production of NO and PGE2, enhanced the protein expression of iNOS and COX2, and promoted the secretion and mRNA level of TNFα via activating the phosphorylation of MAPKs and nuclear translocation of NF-κB in RAW264.7 macrophages.
